# Bayesian inference in genetic parameter estimation of visual scores in Nellore beef-cattle

**DOI:** 10.1590/S1415-47572009005000066

**Published:** 2009-12-01

**Authors:** Carina Ubirajara de Faria, William Koury, Cláudio Ulhôa Magnabosco, Lucia Galvão de Albuquerque, Luiz Antônio Framartino Bezerra, Raysildo Barbosa Lôbo

**Affiliations:** 1 Universidade Federal de Goiás, Campus de Jataí, Jataí, GOBrazil; 2 Brasilcomz Zootecnia Tropical, Jaboticabal, SPBrazil; 3 Embrapa Cerrados, Planaltina, DFBrazil; 4 Universidade Estadual Paulista, Faculdade de Ciências Agrárias e Veterinárias, Jaboticabal, SPBrazil; 5 Universidade de São Paulo, Faculdade de Medicina de Ribeirão Preto, Departamento de Genética, Ribeirão Preto, SPBrazil; 6 Associação Nacional de Criadores e Pesquisadores, Ribeirão Preto, SPBrazil

**Keywords:** morphological traits, threshold model, genetic parameters, Nellore

## Abstract

The aim of this study was to estimate the components of variance and genetic parameters for the visual scores which constitute the Morphological Evaluation System (MES), such as body structure (S), precocity (P) and musculature (M) in Nellore beef-cattle at the weaning and yearling stages, by using threshold Bayesian models. The information used for this was gleaned from visual scores of 5,407 animals evaluated at the weaning and 2,649 at the yearling stages. The genetic parameters for visual score traits were estimated through two-trait analysis, using the threshold animal model, with Bayesian statistics methodology and MTGSAM (Multiple Trait Gibbs Sampler for Animal Models) threshold software. Heritability estimates for S, P and M were 0.68, 0.65 and 0.62 (at weaning) and 0.44, 0.38 and 0.32 (at the yearling stage), respectively. Heritability estimates for S, P and M were found to be high, and so it is expected that these traits should respond favorably to direct selection. The visual scores evaluated at the weaning and yearling stages might be used in the composition of new selection indexes, as they presented sufficient genetic variability to promote genetic progress in such morphological traits.

## Introduction

In Brazilian genetic improvement programs, consideration has been given to growth traits involving the evaluated of weight at different standard ages its gain as criteria for selection in Zebu beef-cattle. The use of these criteria has effectively contributed to the increase in productive performance indices, especially for the Nellore breed. It is noticeable that present day Nellore herds, when it comes to production, are in no way similar to those introduced into Brazil through importation in 1962.

Although, the Nellore population has excelled in body weight, there has been little improvement in finishing precocity and carcass quality. According to Forni *et al.* (2007), weight measurements at certain ages are insufficient to evaluate animal yield and carcass quality at the time of slaughter. However, research involving the estimation of variance components and genetic parameters for traits which are directly involved in the evaluation of carcass quality (longissimus muscle area, backfat thickness, fat thickness and meat tenderness) in Nellore beef-cattle, has shown that there is sufficient genetic variability for such traits, thus paving the way for achieving genetic progress through selection.

Although the carcass quantitative traits, evaluated through ultrasound, allow direct selection for such traits, other indirect measurements can also lead to genetic progress in meat quality. With this in mind, use has been made in various selection programs of information on morphological traits as evaluated by visual scores, with a view to obtaining genetic progress in finishing precocity and carcass maturity and quality in Zebu beef-cattle. Nonetheless, there are only a few studies on estimating genetic and environmental parameters for these visual evaluation traits (Eler *et al.*, 1996; Jorge Júnior *et al.* 2004; Koury Filho, 2005; Kippert *et al.*, 2006; and Faria *et al.*, 2008a).

According to Lôbo *et al.* (2008), the market trend is to seek animals which are more precocious and economically viable, and that remain less time in pastures or feedlots, thereby shortening the production cycle. With this in mind, the National Association of Breeders and Researchers (Associação Nacional de Criadores e Pesquisadores - ANCP) and the company Brasilcomz developed the Morphological Evaluation System (MES, Sistema de Avaliação Morfológica - SAM), which applies modern procedures to collecting data on visual scores for the traits body structure (S), precocity (P), musculature (M) and navel (N), aiming at improving genetic evaluation, and through this, the generation of new selection indexes.

Care must be taken in the genetic evaluation of morphological traits, as data do not present a normal distribution. Souza *et al.* (2000) and Marcondes *et al.* (2005) recommended the use of threshold models as they present higher efficacy in detecting genetic variability when compared to linear models. The threshold model is based on the assumption that classes of observable data are related to the delineation of a normal variable or an underlying continuous scale, which is usually called the liability (Sorensen *et al.*, 1995 and Van Tassell *et al.*, 1998). This liability scale is defined by Gianola and Foulley (1983) as the sum of all those environmental and genetic effects influencing the susceptibility of the trait. Thus, it is assumed that there is a non-observable random variable, associated to the levels in each categorical trait and containing the fixed and random effects.

Therefore, the aim of this work was to estimate variance components and genetic parameters for those visual scores which constitute the Morphological Evaluation System such as body structure (S), precocity (P) and musculature (M) in Nellore beef-cattle, evaluated at the weaning and yearling stages, through the use of threshold Bayesian models.

## Material and Methods

Information was resorted to from visual scores of 5,407 animals evaluated at weaning and 2,649 at the yearling stage, respectively born to 135 and 224 bulls and 2,274 and 2,314 cows. The animals were of the Nellore breed and belonged to herds which took part in the Brazilian Nellore Program of the National Association of Breeders and Researchers (ANCP). The visual scores of MES evaluated in this paper were: body structure (S), precocity (P) and musculature (M). The animals were visually evaluated by means of MES methodology (Koury Filho, 2005), in which body length and animal height are evaluated for S, for P the relationship between rib-depth and limb-height and; for M muscle distribution and length.

Scores from one to six points could be attributed to the tested animals for each morphological variable. When compared to its contemporary group, an animal which is considered intermediate (three or four points) for a specific trait, represents the reference for the classification of those below (one or two points) and above (five or six points) the mean. Data collection for the MES was done by qualified technicians from Brasilcomz.

On applying MES methodology to visual evaluation, the animals from the same management lot were individually evaluated individually by the same technician. Each lot was composed of animals of the same sex and time birth abiding under the same food and sanitary conditions. The entire management lot was taken into consideration, in an attempt to visualize the average profile for each morphological trait evaluated. In this way, visual evaluation was comparative, the score being given to each individual in relation to the others. The distribution of scores as a percentage for each morphological trait is presented Table 1.

Structuring of data files was done by means of the Statistical Analysis System (SAS, 2004) program. In order to guarantee data consistency of morphological traits, the interval in the age of the animals followed a variation from 150 to 270 days in relation to 210 days (age of 7 months) and from 490 to 610 days in relation to 550 days (age of 18 months). GLM and REG procedures from SAS (SAS, 2004) software were used to check environmental effects that influence morphological traits. Weights at 120 days (W120) were included in analyses in order to minimize the effects of selection on visual scores. The individual weight (W120) of 6,169 animals was used, mean and standard deviation being 130 kg and 19 kg, respectively.

Contemporary groups for categorical morphological traits were defined, taking into account the farm, year, season of birth, management lot and the technician responsible for rating lot. As to weight at 120 days, contemporary groups included animals from the same farm, year and season of birth, sex and management lot, on reaching the stipulated age. The effect of season of birth was divided into four classes: animals born in the months of January to March, April to June, July to September and October to December.

Genetic parameters for visual score traits were estimated through two-trait analyses by means of the threshold animal model, using Bayesian statistic methodology with MTGSAM (Multiple Trait Gibbs Sampler for Animal Models) threshold software, developed by Van Tassell *et al.* (1998).

For visual score traits, sex and age of the dam (six classes) were considered as fixed effects, contemporary groups as random effects and the age of animal at the moment of data collection as covariate (linear effect). The complete model can be represented in matricial notation as follows:



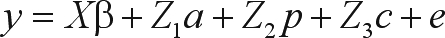


in which *y* is the vector of observations (categorical continuous traits), β is the vector of fixed effects (sex and age of the dam for morphological traits, and contemporary groups for 120-day weight), *a* is the vector of random effects which represent additive genetic values from each animal, *p* is the vector of non-correlated random effects of permanent maternal environmental effects, *c* is the vector of random effects not correlated to contemporary groups, *e* is the vector of residual random effects, and *X*, *Z*_1_, *Z*_2_ and *Z*_3_ are the incidence matrices which link the observations to fixed and random effects: additive genetic direct and non-correlated (maternal permanent environmental and contemporary group), respectively. The relationship matrix included 26,893 animals from the Nellore breed.

Threshold models usually present certain some problems in estimating variance components and predicting genetic values, when there are many levels of fixed effects (Moreno *et al.*, 1997; Varona *et al.*, 1999; and Luo *et al.*, 2002). In a situation like this, the authors recommend assuming these effects as random. However, in order to reach convergence, it is also necessary to have a larger amount of data at each level (Varona *et al.*, 1999). Therefore, the effects of contemporary groups were assumed as being random, and the sex and age of the cow at calving as fixed effects for genetic analyses of visual scores categorical traits.

In the threshold model, it is assumed that the underlying scale presents normal continuous distribution, this being represented as follows:



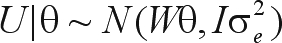


in which *U* is the vector of the base-scale of order *r*, θ' = (β', *a*', *p*', *c*') is the vector of the location parameters of order s with β (defined, from the frequentist view, as fixed effects), and order s with *a*, *p* and *c* (as direct additive genetic random effects and non-correlated to permanent maternal environmental and contemporary groups), *W* is the known incidence matrix of order *r* for s, *I* is the identity matrix of order *r* for *r*; and 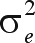 is the residual variance. Given that the liability variate is unobservable, parameterization 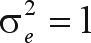 is usually adopted in order to achieve identifiability in likely. Such an assumption is standard in threshold model analysis. The conditional probability that *y*_*i*_ falls into category *j* (*j* = 1, 2, 3, 4, 5, 6), given the vectors β, *a*, *p*, *c* and *t* (*t* = *t*_min_, *t*_1_, ..., *t*_j-1_, *t*_max_) is given by:



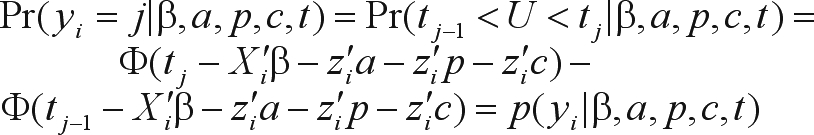


Categorical traits are determined by unobservable continuous variables, in underlying scale, this having fixed threshold initial values, in which *t*_1_ < *t*_2_...< *t*_*j*__-1_, with *t*_0_ = -∞ and *t*_j_ = ∞, where *j* is the number of categories. Observable data are dependent on the underlying variable which is limited between two unobservable thresholds (Gianola and Foulley, 1983).  Therefore, the categories or scores of *y*_*i*_ (morphological traits) for each animal *i* are defined by *U*_*i*_in the underlying scale:



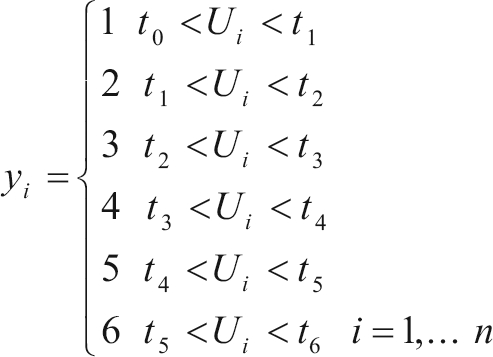


in which *n* is the number of observations. After specifying the thresholds *t*_0_ to *t*_6_, it is necessary that one of the thresholds (from *t*_1_ to *t*_5_) be adjusted to an arbitrary constant. In the genetic analyses for morphological traits evaluated at the yearling stage, it was assumed *t*_1_ = 0, in such a way that the vector of estimable thresholds was defined as:



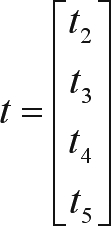


For the two-trait analyses of visual scores evaluated at weaning, another parameterization was adopted (Van Tassell *et al.*, 1998), the residual variance, as well as the residual covariance being estimable among the traits, and assuming that *t*_1_ = 0 and *t*_2_ = 1, in such a way that the vector of estimable thresholds was defined as:



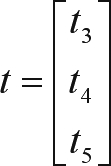


For the two-trait analyses between categorical and continuous ones, according to the Bayesian approach, it was admitted that the initial distribution of genetic random effects, both non-correlated and residual, follows multivariate normal distribution as below:



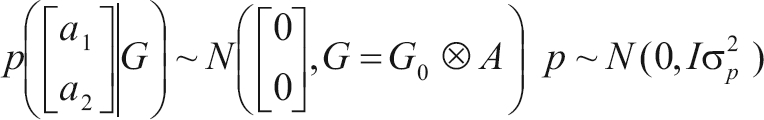




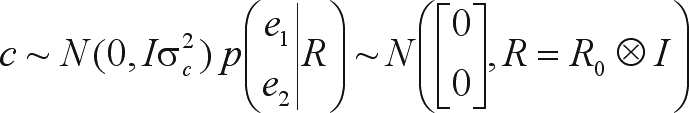


in which *G*_0_ is the matrix of genetic variances and covariances, *R*_0_ is the matrix of residual variances, ⊗ is the direct product operator, *A* is the parentage matrix and *I* is the identity matrix.

Initial distribution of (co)variances was assumed as Inverted Wishart for genetic random effects, and non-correlated and residual in the traits studied, this including mutual covariance. Uniform initial distribution was defined as much for fixed as well as for threshold effects. Parameter *v* is the degree of freedom corresponding to Inverted Wishart distribution, and indicates the degree of initial distribution trustworthiness. In this study, the value of the parameter *v* used was zero, that is, it reflected no degree of knowledge about parameters.

A 1,000,000 total cycle sampler chain, a 300,000 cycle burn-in and a 1,000 cycle thinning interval to obtain 700 initial samples, were used implementation of Gibbs Sampling. Thereafter, the analysis continued, each time 100,000 cycles being added until estimates obtained in the last analysis were equal to the previous one. This criterion was adopted in order to verify whether convergence had really been achieved. Sample analysis, serial correlation and convergence of the Gibbs chain were undertaken with support from the GIBANAL (Van Kaam, 1998) program.

The Monte Carlo error was estimated by calculating the variance in samples of genetic parameters of traits and dividing this variance by the number of samples (Van Tassell *et al.*, 1998). Thus, the square root of this value is an approximation of the standard deviation of the Monte Carlo error associated to Gibbs chain length.

The solutions of genetic values obtained for each animal were transformed into a probability scale through the SAS PROBNORM (SAS, 2004) function, which resulted in EPDs (expected progeny differences) expressed as a percentage (%). The PROC CORR procedure from SAS (SAS, 2004) software was applied to verify rank or Spearman rank-order correlations.

## Results and Discussion

Descriptive statistics of variance components and genetic parameters of morphological traits, evaluated by MES methodology at weaning, are presented in Table 1. It was noted that heritability estimates found for body structure (S), precocity (P) and musculature (M) were high, thus, it is expected that these traits should respond positively to direct selection.

The estimates of heritability for S (0.68), P (0.65) and M (0.62) indicate high genetic variability for visual scores, which is explained by the differences in biotypes or morphological types existent in Nellore cattle. It is important to point out that the application of MES methodology to morphological evaluation is novel, and thus selection for the visual scores S, P and M is also relatively recent in Brazil.

A similar result for P heritability was encountered by Koury Filho (2005), in which the estimate was 0.63. According to the author, estimated heritability for both P and M was higher than that obtained for body weight and consequently, the expected responses for directing the selection of such traits might be superior to those for body weight.

It is important to emphasize that the maternal additive genetic random effect of visual scores evaluated at weaning was not included in the model due to the difficulty in attaining Gibbs chain convergence, when applying threshold models to an animal model. Other authors (Eler *et al.*, 1996; Jorge Júnior *et al.*, 2004) reported low estimates for heritability coefficients calculated for the maternal genetic effect, when considering the traits conformation (C), precocity (P) and musculature (M) in Nellore beef-cattle. According to Forni *et al.* (2007), no significant changes are expected in visual scores derived from the maternal genetic effect when selecting. Thus, non inclusion of the maternal additive genetic effect did not interfere in the results of the estimates herein presented.

Nevertheless, models which do not take maternal effects into consideration may lead to higher estimates of direct additive genetic variance, and consequently, higher estimates of heritability (Meyer, 1992). This fact could explain high heritability in S, P and M. Thus, the non-correlated permanent maternal environmental effect was included in the model on purpose, so as to avoid this possible overestimation of direct additive genetic variance, as around 80% of the cows had more than one calf with visual scores data, this permitting the inclusion of this effect in analyses.

Furthermore from Table 2, it can be seen that the proportion of permanent maternal environmental random effect was less than 3%, as indication of its low variance. The contrary was presented by Forni *et al.* (2007), when evaluating data from visual scores on in Nellore beef-cattle. They found that the proportion of this same effect on phenotypical variance of these traits of 14%. According to the latter heritability estimates were 0.12 for C, 0.15 for P and 0.12 for M, when considering linear models on estimating genetic parameters.

The estimates of variance components and genetic parameters for morphological traits evaluated by the MES methodology at the yearling stage are presented in Table 3. It can be seen that estimates (means) of heritability were high for S (0.44), P (0.38) and M (0.32), but lower when compared to those obtained at weaning. These results corroborate findings encountered in the literature on Nellore cattle (Eler *et al.*, 1996; Koury Filho, 2005; Kippert *et al.*, 2006; Forni *et al.*, 2007 and Faria *et al.*, 2008b), to the effect that visual scores, evaluated in yearlings, respond to direct selection.

Regarding the application of Bayesian threshold models, it can be seen from in Tables 2  and 3 that the mean, mode and median of the estimates of genetic parameters were similar for all of the morphological traits evaluated at both weaning and the yearling stage. Such results indicate that Gibbs chain convergence was accomplished, there being posterior marginal distribution of (co)variances components tending to normal distribution. It is important to note that symmetry in estimating central tendency measurements is indicative of sampling chain convergence, as well as accurate analysis (Silva *et al.*, 2005). However, it is not necessary for estimates of central tendency measurements of (co)variance components to be similar, as Inverted Wishart distribution of posterior marginal densities of (co)variances components is expected (Sorensen *et al.*, 1995; and Van Tassell *et al.*, 1998).

The Monte Carlo error represents a mistake in parameter estimation due to the number of samples used in the Gibbs chain (Sorensen *et al.*, 1995). According to Van Tassell and Van Vleck (1996), the Monte Carlo error is inversely proportional to the Gibbs chain-length, knowledge of which being extremely important for evaluating whether the implementation of Gibbs Sampling was adequate or not to generate the posterior means of (co)variance components marginal distribution. It was verified from Tables 2  and 3 that the Monte Carlo error was too small for all of variance components and genetic parameters, thus indicating that the Gibbs chain-length was sufficient to obtain accurate estimates of posterior means. The Monte Carlo error is considered small when its value, added to the mean estimate of heritability coefficient posterior distribution, does not alter the value of this estimate, when rounded to the second decimal place of heritability. Therefore, it may be inferred that application of the Bayesian threshold model was efficient enough to obtain estimates of genetic parameters for categorical morphological traits.

From Table 3, it can be observed that the genetic correlations between weight at 120 days and visual scores were 0.94 for S, 0.62 for P and 0.72 for M. Generally, it is expected that direct selection from visual scores leads to a positive correlated response to standard 120-day weight. Nonetheless, the important point to note is that at the yearling stage the estimate of genetic correlation for S was the highest, as was expected, since the body structure (S) trait is related to animal-size, wich leads one to infer that the two traits (body structure and weight) are significantly influenced by the same gene groups. The same did not occur with M, in wherein the estimate of genetic correlation was 0.72, thus even lower than P (0.62), also as expected, since the P trait is set on a relationship between rib depth and limb height and thus a lower association to weight (W120) than S. These results also place in evidence that MES methodology of visual evaluation was efficient in defining body structure, precocity and musculature, thereby accurately identifying the specifications of each trait.

Nevertheless, as observed, the estimates of genetic correlations between the standard weight at 120 days (W120) and visual scores evaluated at weaning were high for all morphological traits (Table 2). Based on these results, one may infer that at weaning the morphological traits of S, P and M may not be appropriately expressed genetically, and mutual differences not entirely placed in evidence. However, the inclusion of visual scores as selection criteria is necessary, at least in two phases of the animal's life, at weaning and the yearling stage.

Faria *et al.* (2008a) came to the same conclusions, in Nellore beef-cattle, evaluated for musculature (M), body structure (S) and conformation (C). The authors concluded that, although morphological traits evaluated at weaning presented high heritability estimates, they might possibly not be well defined at this age, and mutual genetic differences might be better detected at a later age. Koury Filho (2005) considers a single-moment decision as premature for evaluating the animals, for if evaluation at weaning is very interesting through not involving pre-selection, at the yearling stage, morphological traits better express the direct individual genetic potentiality.

In Table 4, descriptive statistics of EPDs (expected progeny differences) for morphological traits evaluated with MES methodology at weaning and the yearling stage in of Nellore beef-cattle appear. It is noticeable that EPDs are important tools for aiding the breeder when taking decisions concerning the selective process in a herd. In Table 4, it can be seen that the means of EPD estimates for all the traits evaluated are below 50%, which indicates little or no selection of such traits in the Nellore herds evaluated. Under such conditions, and taking into account the high heritability estimates in S. P and M, it is possible to infer that there is a quick response to direct selection for such traits. It was also verified (Table 4) that genetic variability is similar for all visual score traits (see maximum and minimum values). Similar results were found by Faria *et al.* (2008b), although the morphological traits evaluated were musculature (M), physical structure (S) and conformation (C), when considering the application of threshold models to field data in Nellore cattle.

Rank or rather Spearman rank-order correlations of EPDs (expected progeny differences) in morphological traits evaluated through MES methodology at weaning and the yearling stage, are presented in Table 5. It can be seen that traits evaluated at weaning were higher (above 90%) when compared to those among yearlings. Therefore, it might be inferred that genetic differences among the visual scores are more in evidence when evaluated at the yearling stage.

Rank correlations among visual scores evaluated at different ages were 0.78 for S, 0.61 for P and 0.66 for M. These results show that classification or ranking of the animals changed when a certain morphological trait was evaluated at different ages (weaning and yearling). It was noted that for S, the higher rank correlation (0.78) reflects the intense genetic association of this trait with body weight, and that, no matter the age at visual evaluation, genetic expression become evident. The same was not the case with P and M, in which genetic differences were better presented at the yearling stage.

In conclusion, visual scores from the Morphological Evaluation System, evaluated at weaning and the yearling stage, could be used in composing new selection indexes, as they presented sufficient genetic variability to promote genetic progress in such morphological traits.

## Figures and Tables

**Table 1:** Distribution (%) of scores for the traits body structure (S), precocity (P) and musculature (M) evaluated by means of MES methodology at weaning (W) and the yearling stage(Y).

Trait	Number	Mean^a^	Scores					
			One	Two	Three	Four	Five	Six
S_W_	5,407	3.84	8.5	12.2	19.6	22.7	20.2	16.8
P_W_	5,405	3.96	7.1	12.2	16.2	23.3	23.9	17.3
M_W_	5,407	3.90	8.2	12.6	17.3	21.1	24.0	16.8
S_Y_	2,649	3.88	5.0	13.5	21.4	24.5	19.1	16.5
P_Y_	2,648	3.86	6.8	14.4	18.3	23.3	20.7	16.5
M_Y_	2,649	3.69	6.9	16.2	22.4	21.7	20.6	12.2

^a^ 
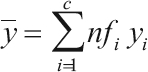
.

**Table 2:** Estimates of genetic parameters for the traits body structure (S), precocity (P) and musculature (M) evaluated by MES methodology at weaning, and obtained through Bayesian two-trait analyses with a threshold animal model.

Genetic parameter	Descriptive statistics				
	Mean	Mode	Median	CR	MCE
	Body structure (S)				
σ^2^_a_	2.81	2.82	2.81	2.36-3.27	0.0084
σ^2^_p_	0.19	0.19	0.19	0.19-0.19	0.0000
σ^2^_c_	0.33	0.30	0.32	0.21-0.49	0.0028
σ^2^_e_	0.74	0.73	0.74	0.56-0.91	0.0032
h^2^	0.68	0.68	0.68	0.63-0.74	0.0010
p^2^	0.04	0.04	0.04	0.04-0.04	0.0000
c^2^	0.08	0.08	0.08	0.05-0.11	0.0020
r_g_	0.92	0.92	0.92	0.90-0.94	0.0003

	Precocity (P)				
σ^2^_a_	2.41	2.48	2.42	1.98-2.83	0.0085
σ^2^_p_	0.12	0.12	0.12	0.03-0.21	0.0016
σ^2^_c_	0.16	0.15	0.15	0.07-0.30	0.0020
σ^2^_e_	0.97	0.98	0.97	0.90-1.03	0.0012
h^2^	0.65	0.65	0.65	0.60-0.70	0.0008
p^2^	0.03	0.03	0.03	0.00-0.05	0.0004
c^2^	0.04	0.04	0.04	0.02-0.07	0.0004
r_g_	0.94	0.94	0.94	0.91-0.96	0.0004

	Musculature (M)				
σ^2^_a_	2.07	2.08	2.07	1.94-2.19	0.0024
σ^2^_p_	0.07	0.00	0.07	0.00-0.20	0.0026
σ^2^_c_	0.25	0.24	0.25	0.15-0.40	0.0023
σ^2^_e_	0.91	0.90	0.91	0.85-0.96	0.0010
h^2^	0.62	0.62	0.62	0.59-0.65	0.0006
p^2^	0.02	0.00	0.02	0.00-0.05	0.0007
c^2^	0.07	0.07	0.07	0.04-0.11	0.0006
r_g_	0.99	0.99	0.99	0.99-0.99	0.0001

σ^2^_a_ additive genetic variance; σ^2^_p_ maternal permanent environmental variance; σ^2^_c_ contemporary group variance; σ^2^_e_ residual variance; h^2^ heritability; p^2^ proportion of phenotypic variance due to permanent maternal environmental group; c^2^ proportion of phenotypic variance due to contemporary group; r_ge_ genetic correlations between the weight at 120 days and visual scores; CR credibility region 95%; MCE Monte Carlo error.

**Table 3:** Estimates of genetic parameters for the traits body structure (S), precocity (P) and musculature (M) evaluated by MES methodology at the yearling stage, and obtained through Bayesian two-trait analyses with a threshold animal model.

Descriptive statistics	Genetic parameters				
	σ^2^_a_	σ^2^_c_	h^2^	c^2^	r_g_
	Body structure (S)				
Mean	1.24	0.56	0.44	0.19	0.94
Mode	1.20	0.53	0.44	0.19	0.94
Media	1.21	0.54	0.44	0.19	0.94
Credibility region (95%)	0.88-1.74	0.30-0.91	0.35-0.52	0.11-0.28	0.88-0.98
Monte Carlo error	0.0006	0.0004	0.0001	0.0001	0.0001

	Precocity (P)				
Mean	0.83	0.31	0.38	0.14	0.62
Mode	0.82	0.27	0.40	0.14	0.62
Media	0.83	0.29	0.39	0.14	0.62
Credibility region (95%)	0.54-1.15	0.14-0.59	0.29-0.47	0.07-0.23	0.51-0.72
Monte Carlo error	0.0046	0.0034	0.0013	0.0012	0.0016

	Musculature (M)				
Mean	0.70	0.45	0.32	0.20	0.72
Mode	0.67	0.40	0.32	0.21	0.72
Media	0.69	0.43	0.32	0.20	0.72
Credibility region (95%)	0.44-1.04	0.24-0.77	0.23-0.42	0.12-0.30	0.60-0.82
Monte Carlo error	0.0046	0.0042	0.0014	0.0014	0.0016

σ^2^_a_ additive genetic variance; σ^2^_c_ contemporary group variance; h^2^ heritability; c^2^ proportion of phenotypic variance due to contemporary group; r_ge_ genetic correlations between the weight at 120 days and visual scores.

**Table 4:** Descriptive statistics of EPDs (expected progeny differences) for the traits body structure (S), precocity (P) and musculature (M), evaluated by MES methodology, at weaning (W) and the yearling stage (Y).

Trait	Mean	Mode	Minimum	Maximum
S_W_	49.1	12.1	25.0	75.0
P_W_	48.8	11.5	25.0	75.0
M_W_	49.7	11.1	25.0	75.0
S_Y_	49.4	8.6	25.0	74.8
P_Y_	46.0	6.7	25.4	73.5
M_Y_	47.0	6.4	25.3	73.7

**Table 5:** Rank correlation among the EPDs (expected progeny differences) of morphological traits evaluated by the MES methodology at weaning (W) and the yearling stage (Y).

	S_W_	P_W_	M_W_	S_Y_	P_Y_	M_Y_
S_W_	1	0.92	0.99	0.78	0.57	0.67
P_W_		1	0.92	0.77	0.61	0.68
M_W_			1	0.81	0.55	0.66
S_Y_				1	0.61	0.74
P_Y_					1	0.87
M_Y_						1
